# Community views on the secondary use of general practice data: Findings from a mixed‐methods study

**DOI:** 10.1111/hex.13984

**Published:** 2024-02-15

**Authors:** Annette J. Braunack‐Mayer, Carolyn Adams, Alberto Nettel‐Aguirre, Belinda Fabrianesi, Lucy Carolan, Justin Beilby, Felicity Flack

**Affiliations:** ^1^ Australian Centre for Health Engagement, Evidence and Values (ACHEEV), School of Health and Society, Faculty of the Arts, Social Sciences and Humanities University of Wollongong Wollongong New South Wales Australia; ^2^ Australia Health Services Research Institute University of Wollongong Wollongong New South Wales Australia; ^3^ Macquarie Law School Macquarie University Sydney New South Wales Australia; ^4^ National Institute for Applied Statistics Research Australia University of Wollongong Wollongong New South Wales Australia; ^5^ School of Health and Society University of Wollongong Wollongong New South Wales Australia; ^6^ Population Health Research Network University of Western Australia Perth Western Australia Australia

**Keywords:** community views, data linkage, data sharing, ethical issues, general practice data, research

## Abstract

**Introduction:**

General practice data, particularly when combined with hospital and other health service data through data linkage, are increasingly being used for quality assurance, evaluation, health service planning and research. In this study, we explored community views on sharing general practice data for secondary purposes, including research, to establish what concerns and conditions need to be addressed in the process of developing a social licence to support such use.

**Methods:**

We used a mixed‐methods approach with focus groups (November–December 2021), followed by a cross‐sectional survey (March–April 2022).

**Results:**

The participants in this study strongly supported sharing general practice data with the clinicians responsible for their care, and where there were direct benefits for individual patients. Over 90% of survey participants (*N* = 2604) were willing to share their general practice information to directly support their health care, that is, for the primary purpose of collection. There was less support for sharing data for secondary purposes such as research and health service planning (36% and 45% respectively in broad agreement) or for linking general practice data to data in the education, social services and criminal justice systems (30%–36%). A substantial minority of participants were unsure or could not see how benefits would arise from sharing data for secondary purposes. Participants were concerned about the potential for privacy breaches, discrimination and data misuse and they wanted greater transparency and an opportunity to consent to data release.

**Conclusion:**

The findings of this study suggest that the public may be more concerned about sharing general practice data for secondary purposes than they are about sharing data collected in other settings. Sharing general practice data more broadly will require careful attention to patient and public concerns, including focusing on the factors that will sustain trust and legitimacy in general practice and GPs.

**Patient and Public Contribution:**

Members of the public were participants in the study. Data produced from their participation generated study findings.

**Clinical Trial Registration:**

Not applicable.

## 1 INTRODUCTION

General practice data, particularly when combined with hospital and other health service data through data linkage, are increasingly being used for quality assurance, evaluation, health service planning and research.^1^, ^2^, ^3^ Using general practice data is particularly important in countries where general practitioners (GPs) are the first and principal source of health care for most people.^3^

Although there is broad public support for the secondary use of health data,^4^, ^5^, ^6^, ^7^, ^8^ there are good reasons to question whether this support extends to general practice settings. GP–patient relationships may be very personal and longstanding and the general practice health record can capture a large amount of information about patients.^9^ There is also the potential for multiple angles on patients' lives: GPs often care for, or at least record information about, more than one generation of a family. These factors combine to amplify patients' and GPs' concerns about sharing patient data.^1^

The experience in the United Kingdom with *care.data*—a National Health Service initiative to extract data from general practice records using an opt‐out model—suggests that addressing public concerns about the secondary use of general practice data requires more than simply complying with the law.^10^ The decision by the National Health Service to collect information from general practices using an opt‐out approach and to make this information available in aggregated form was lawful.^11^ However, the public and professional outcry against the initiative was so overwhelming that implementation of the scheme was initially suspended and then finally abandoned. Clearly, mere legal authority was necessary but not sufficient to successfully implement *care.data*; social approval or a ‘social licence’ was also necessary to meet societal expectations about the responsible use of health data.

Muller et al.'s recent narrative review^12^ brings together the essential components of a social licence, specifically related to data‐intensive health research, defining social licence as:

… the non‐tangible societal permission or approval that is granted to either public or private researchers and research organisations. This allows them to collect, use, and share health data for the purpose of health research by virtue of those activities being trustworthy, by which is meant trusted to be in line with the values and expectations of the data subject communities, stakeholders, and the public.^12^

Adams et al. have developed a model of social licence, specifically in the context of sharing administrative data for health research, based on an analysis of the social licence literature and founded on two principal elements: trust and legitimacy.^13^ In this model, trust is founded on research enterprises being perceived as reliable and responsive, including in relation to privacy and security of information, and having regard to the community's interests and well‐being.

Transparency and accountability measures may be used to demonstrate trustworthiness and, as a consequence, to generate trust. Transparency involves a level of openness about the way data are handled and used as well as about the nature and outcomes of the research. Adams et al. note that lack of transparency can undermine trust.^13^ They also note that the quality of public engagement is important and that simply providing information is not sufficient. While this is one element of transparency, other elements such as accountability and collaboration are also part of the trusting, reflexive relationship necessary to establish and support social licence.

The second principal element, legitimacy, is founded on research enterprises conforming to the legal, cultural and social norms of society and, again, acting in the best interests of the community. In diverse communities with a range of views and interests, it is necessary to develop a broad consensus on what amounts to the common good through deliberative and collaborative processes.^14^

Social licence cannot be assumed. It must be built through public discussion and engagement to avoid undermining the relationship of trust with health care providers and confidence in the confidentiality of health information.^13^

The existing literature on social licence and the sharing of health data for research is largely focused on sharing administrative data, particularly administrative data in the hands of government, for research. In this realm, sharing health data for secondary purposes is heavily regulated including through specific privacy and data collection legislation. In addition, government departments are subject to legal duties of confidentiality and an overriding duty to act in the public interest.

In Australia, general practices are private sector organisations and the health data held by GPs are not as heavily regulated. GPs are, however, subject to the common law duty of confidentiality and to privacy legislation in those jurisdictions with such legislation in place. As the technical capacity to share and link the data in diverse general practice records develops, it is important to consider the regulatory environment to ensure that it supports the conditions necessary for the development of a social licence to share general practice data.

The literature in relation to sharing general practice data for secondary purposes is more limited.^10^, ^15^, ^16^, ^17^, ^18^, ^19^ Thus, in this study, we set out to explore community views specifically on sharing general practice data for secondary purposes, including research. We explored community concerns with sharing such data for research and the conditions that could alleviate these concerns. The findings reported in this paper are part of a larger study of community understandings of and attitudes towards the use of general practice data for secondary purposes.

## 2 METHODS

We used a mixed‐methods approach with focus groups (November–December 2021), followed by a cross‐sectional survey (March–April 2022). We chose focus groups to develop an understanding of shared community views about the secondary use of general practice data, expecting that participants would be able to build on each other's experiences and understandings. We followed the focus groups with an online panel survey because it provided a rapid and cost‐effective way to explore a topic in which practice and understanding are rapidly evolving.^20^ We also wanted to test whether the range of views expressed in the focus groups would be reflected in a larger sample of the Australian population. Moreover, the panel composition was created with breadth and representativity in mind. We also weighted the sample responses with weights based on Australian Bureau of Statistics (ABS) census data to adjust for over‐ or underrepresentation of respondents.

### 2.1 Recruitment

We employed an experienced Australian market research company, McNair yellowSquares,^21^ to recruit a sample of participants from their opt‐in online panel for both the focus groups and survey.

For the focus groups, McNair yellowSquares were asked to secure a diverse sample with respect to gender, age, residential location, educational qualifications, employment and cultural background. We invited panel members 18 years and older and excluded people currently or previously employed in a general practice setting. We recruited participants to one female‐only group (>25 years), one male‐only group (>25 years), one mixed‐gender group (>25 years) and one young persons' group (18–25 years). In recognition of their time, focus group participants received a $90 voucher.

For the survey, McNair yellowSquares aimed to recruit an opt‐in sample of 2500 participants, selected to be nationally representative by age, gender and location. Survey participants received an incentive of up to $2.

### 2.2 Design

#### 2.2.1 Focus groups

There is good evidence that community members may not be familiar with data‐intensive research.^5^, ^22^, ^23^, ^24^, ^25^ With this in mind, we included an element of information provision in the focus groups to better support participants to understand, navigate and respond to the topic in a group setting. We developed four case‐based scenarios to prompt participants to explore the types of information captured in general practice records and with whom and why general practice data are shared (Appendix S1). We piloted the discussion guide with a convenience sample of six adults. We made minor amendments to the discussion guide after the pilot. Appendix S2 COREQ (Consolidated Criteria for Reporting Qualitative Research)^26^ provides more detail about the methods.

#### 2.2.2 Survey

The online survey (Appendix S3) adapted questions from pre‐existing tools with new questions and insights from the literature^15^, ^17^, ^23^, ^27^, ^28^, ^29^, ^30^, ^31^, ^32^, ^33^, ^34^ and the focus group findings. It included two short videos that briefly explained the content of a general practice health record and how this information can be shared with other people and organisations. We also collected sociodemographic characteristics, health status and general practice attendance for participants. A single open‐ended question at the end of the survey invited additional comments. The SURGE (The SUrvey Reporting GuidlinE)^35^ provides more detail (Appendix S4).

### 2.3 Data analysis

#### 2.3.1 Focus groups

Focus groups were audio‐recorded and transcribed verbatim by a professional transcription service Scribefire.^36^ Two researchers (L.C. and A.J.B.‐M.) coded transcript one, to develop and agree on a structure. The coding tree focused on awareness and understanding of the content of GP and hospital records; support for sharing and linkage (including perceived benefits); concerns about sharing and linkage; and conditions and controls on sharing and linkage. We summarised group dynamics and content of group discussions, with particular attention to the ways in which each group was similar or different.

#### 2.3.2 Survey

We weighted data based on ABS census data^37^ and used R Project for Statistical Computing^38^ to analyse the data. Only completed survey data were analysed. To support population inference, we analysed the survey data using poststratification with gender, age, place of residence and highest educational attainment weights. We used the 2016 ABS census data^39^ to obtain the Australian population characteristics of gender, age, state and education and calculate the survey weights based on the realised sample characteristics after combining categories with small sample counts. We used the rake method^40^ because not all possible crossings of all possible levels of the variables chosen were observed in the data. All results in this paper except for participant demographics are obtained using such weights. Raw proportions are only reported on participants' demographic information.

## 3 RESULTS

### 3.1 Demographics

In total, 22 participants attended the four focus groups. There were equal numbers of female and male participants, with most aged 18–29 years (*n* = 10), all but seven with postsecondary education, residing in New South Wales or Victoria (*n* = 14) and working full time (*n* = 11) (Appendix S5).

The survey was completed by 2604 participants. Full demographic information about participants is presented in Appendix S6.

### 3.2 Views on sharing general practice data

#### 3.2.1 With whom should general practice data be shared and for what purposes?

We asked survey and focus group participants with whom and for what purposes it would be acceptable to share information from their general practice record. In the survey, we explained that the GP and everyone with whom information was shared would do everything that they could to minimise privacy risks but that it was still possible that people could be identified from some of the information shared about them.

**Figure 1:**
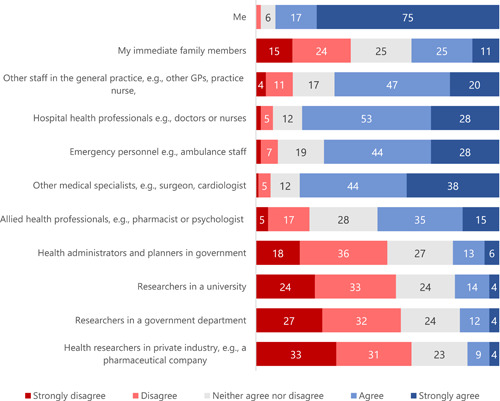
Participant responses to the question ‘To what extent do you agree with the information from your general practice record being shared with the following people?’ (%). GP, general practitioner.

**Figure 2:**
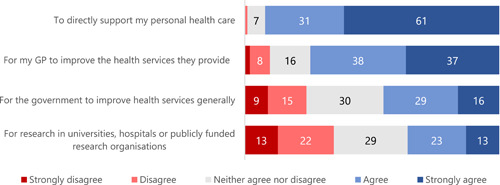
Participant responses to the question ‘To what extent do you agree with information from your general practice record being shared for the following reasons?’ (%). GP, general practitioner.

Over 90% of survey participants were willing to share their general practice information to directly support their health care (Figures 1 and 2). Between 50% and 82% were also willing to share the information with other health care providers, with the greatest support for sharing with other medical specialists and the least support for sharing with allied health staff such as pharmacists (Figure 1). Overall, there was far less support for sharing general practice information with health administrators, planners and researchers (Figure 1) but, paradoxically, about half of the participants thought that it would be acceptable to share information for purposes related to health service planning or research (Figure 2). For many of these questions, around a quarter of all respondents were undecided.

**Figure 3:**
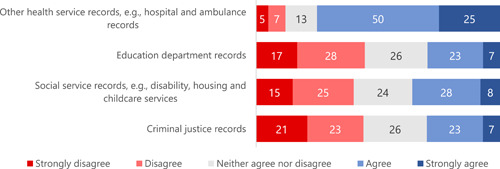
Participant responses to the question ‘To what extent do you agree with the information from your general practice record being linked with information about you from the following sources for specific research purposes?’ (%).

We also asked participants about linking information from their general practice record with records from other health services and with information from outside the health system for specific research purposes. We prefaced these questions with a short video about the protections in place when personal data are shared and linked for research, and we also reminded participants that it was still possible that people could be identified from some of the information shared about them. There was broad support for linking general practice records with other health service records (75% agreeing or strongly agreeing) and much less support for linking general practice data with educational, social services or criminal justice data (30% to 36%) (Figure 3).

The breadth of views in the survey was mirrored in the focus groups. Focus group participants emphasised the potential for societal benefit, noting that large data sets could be used to improve health status and services and contribute to well‐being in society more generally. Some participants also described specific benefits in terms of additional staffing, better health facilities and more effective interventions and treatments. Linked data sets, in particular, could provide a more comprehensive picture of the population.

P4 (29 years): … If the researcher is able to use our information in a way that both us and the researcher are contributing to a small step towards the greater good, that's similar to what P2 says. Use it properly because who knows what the future might bring? We may be working towards the future with pills that can cure diabetes, pills that can cure heart disease, cancer, everything. Just to be a part of that tiny bit of future of mankind. (All female FG1)

Some participants personalised these benefits and suggested that data sharing and linkage could improve the care that they received from their GP.

P5 (female 24 years): I think it should be used for research purposes that further benefit patients and work to improve their healthcare, either on an individual level or on a more systemic level. (Mixed‐gender youth FG4)

### 3.3 What conditions should be placed on sharing general practice data for secondary purposes?

Although there was broad support amongst focus group participants for sharing general practice data for defined purposes, the participants wanted to place strict conditions on sharing data, linked to specific concerns.

#### 3.3.1 Concerns

Many participants were concerned about privacy breaches and the possibility of discrimination, noting, in both cases, that the risk increased as more data sources were linked.

P1 (43 years): I don't know about domestic violence because if they use the whole history I think someone can identify who that person is. I'm not really sure if all of this should be made transparent, because if you take the whole history and put it there, then someone can identify with all these conditions, ‘Okay. I know this person'. That's what might happen. (All female FG1)

P4 (52 years): I agree. I don't see the relevance, unless they're looking at people's wealth and demographics due to their wealth. If they live out in a certain area and their education is poor or something, are they going to be discriminated against? Even though there's anonymity, like the other guys are saying—if the data gets in the wrong hands, then certainly, you're likely to be discriminated against based on—your health might be poor if you can't get the right food, you can't get this and that, or the right education. (All male FG2)

Participants were also concerned about the trustworthiness of the people and institutions who would use their data. Some participants were worried that their personal information simply could not be kept safe, citing previous breaches, particularly of government records, as evidence that data could not be protected adequately.

P4 (male 65 years): You're trusting the safety of their computer system in the surgery to keep it secure. (Mixed‐gender FG3)

P7 (male 19 years): For me, my address is very important to me—it's where I live, and my area where I am. … The census last year got hacked, and people had names, their addresses in all the censuses of themselves run by the government. It was hacked before. (Mixed‐gender youth FG4)

Some participants doubted the competence of the people who would have access to data, particularly if they thought that these people might not have the expertise to use or interpret data wisely. Some were concerned that GPs were not necessarily trained to interpret statistics; others worried that the people interpreting data might lack the medical expertise needed to draw sound conclusions.

P6 (male 20 years): You don't necessarily know the medical expertise of the people in the government department, so whether they actually are using the information to do what should be done ideally, whereas previously it was people who at least were in the medical profession, so you would assume that they would have some sort of knowledge that they could use to actually understand the statistics that were given and the information that they collected. (Mixed‐gender youth FG4)

Finally, participants expressed concerns about the potential for profit motives to influence data custodians and users. Several participants were not in favour of sharing general practice data if insurance companies, GPs or researchers could profit financially from data sharing.

P1 (59 years): I think, maybe, sometimes information might be misused. Say, for example, insurance companies might turn around and say, ‘Look, we've got this report from the government saying that 50 percent of people who live in this area have experienced this problem.’ We might jack up the premiums just to cover ourselves, just in case a claim's been made. That's high‐level. (All male FG2)

P5 (female 24 years): I think it should be used for research purposes that further benefit patients and work to improve their healthcare, either on an individual level or on a more systemic level. I don't think it should be used for other reasons, like just for the GP practice, to get them different funding from the government or things like that. (Mixed‐gender youth FG4)

#### 3.3.2 Conditions on sharing data

To address the concerns above, we asked both survey and focus group participants about the conditions that they would want to see met before data could be shared.

First, both survey and focus group participants wanted *identifying information (names, addresses and dates of birth) to be removed* before the information was shared. In the survey, 84% of participants wanted this to occur (Appendix S7). Most focus group participants said that, once identifying information was removed, they would be very comfortable with linking data with other sources:

P4 (29 years): I really think with the anonymity of all the information, it doesn't really bother me. I don't really know what they share about me anyway, even if they are, so my name is not there.

P2 (59 years): Yeah. No one knows you. That's right. It's anonymous … Because it's anonymous it's an easy way to gather a lot of information quite quickly and literally quite unobtrusively too. Because of the anonymity, I think everyone is protected. (All female FG1)

A small number of focus group participants understood that linking general practice data with other sources increased the possibility that patients could be reidentified from their data, even though names, dates of birth and addresses had been removed:

P2 (female 24 years): … that could kind of skid off into personal information that, while not identified, if you know that community, you could possibly identify them.

Facilitator: Because you've got more information about them, is that it?

P2: Because you know the hospital, the ambulance, if you go to a physio or stuff like that—it's easier if that information is accessible to the public, and while deidentified, it's plausible and possible. (Mixed‐gender youth FG4)

Second, participants wanted *transparency* with respect to the information collected, held and shared. Most survey participants wanted to know when the information was being shared, with whom and why (84%, 86% and 85% agreeing or strongly agreeing, respectively). One focus group participant suggested a campaign to raise awareness about data use and its benefits, including why it would be shared and with whom.

P1 (male 28 years): It doesn't make a difference in my perspective. You can do it. The thing is, it's good to opt in, but before that I think it should be some sort of campaign or awareness should be made, just for telling people that, ‘We have used your data to do these good things, and we are trying to improve it,’ so that people can know what sort of things are being done with the data, so that they will be further happy to opt in, things like that.

P4 (male 65 years): Maybe, P3, that might speak to you. If you could see some of the results of having this data, this information, what the results were … If you could see what comes out of having access to this sort of data, the differences in treatment and the provision of facilities, maybe that might be more encouraging to you. I don't know. Give you a reason why you're doing it. This is the reason. (Mixed‐gender FG3)

There was strong support in the focus groups for *consent* to data sharing, regardless of who the data would be shared with and the purpose of sharing. At the point at which participants first mentioned consent, the moderator provided a brief description of four approaches to consent: opt‐in, opt‐out, waiver of consent with information provided and waiver with no information provided. When participants were asked which approach they preferred, almost all chose opt‐in consent. They explained that informed consent provided recognition that patients' data were being shared and it also offered a vehicle through which transparency would be assured.

P1 (59 years): I agree. I think you need to have an informed discussion with your GP and the GP needs to outline what information's going to be shared and for what reasons, and we're not medical people, and we need to understand—well, I certainly do want to understand—what am I going to share? So, I need to make an informed decision. That's why opt‐in is a no‐brainer for me. (All male FG2)

Although supportive of opt‐in consent, some participants noted that it might not be practicable for data collection, and others were concerned that patients would feel pressured to opt‐in if their GPs were asking them to consent.

P2 (37 years): I think I agree with the opt‐in option, but often the cases what we have seen is the consent waiver, so basically, the GPs just get a blanket consent waiver from the patients—whoever is visiting the GP—and that's what the reality is. I don't think the GPs have time or the patience to actually explain to each and every patient about the opt‐in process. (All male FG2)

After participants had discussed which consent approach they preferred and why, the moderator explained that, for very large data sets, opt‐in consent models can make the collection of patient data held in general practice records impracticable and their use untrustworthy. The focus groups varied considerably with respect to their response to this information: in two focus groups, no one changed their minds, in another focus group, all participants changed from opt‐in to waiver of consent and in the fourth, most participants shifted towards a waiver of consent. Participants who maintained their support for opt‐in consent placed a high value on privacy and choice. Those who shifted to supporting a waiver of consent tended to make their support conditional on anonymisation of data and greater understanding of who data would be shared with and for what purposes.

P2 (59 years): Yeah. I wouldn't have a problem if, again, I knew that the practice I was going to did that, as long as I was made aware. I didn't need to be asked every time but again how do you know that? Then if you choose to not go there, that's your choice, but how do they make all their patients aware that it's a sharing information practice? (All female group FG1)

P4 (male 65 years): My overriding feeling is that if it's completely anonymous information, there is no way that anybody can be personally identified, does it matter who has access to it? (Mixed‐gender FG3)

This range of views was reflected in the survey. Although most participants (over 80% broad agreement or strongly agreeing) were confident that their GP would take care of the information in their health record, there was no consensus with respect to whether their GP should be able to share their health information without informing them. Thirty percent were willing to allow the GP to make this decision, almost half the survey participants (49%) did not want their GP to do this and 20% were undecided.

Finally, many focus group participants wanted *regulations and policies* to control what and when data could be shared, with whom and for what purposes. They wanted systems in place that would keep data secure, avoid data breaches and ensure that individuals were not identified. They also wanted ways to limit access to data to trusted entities. For most participants, these controls were a condition of their support.

P2 (59 years): I think it would have to be quite well‐regulated. I have absolutely no problem with putting all the information out there, but it can't just be up‐for‐grabs for anybody. It's got to be like Joe Blow can't just get this information. It has to literally be vetted as to who actually gets it. I think it would hugely, hugely advantageous … There's a lot of trust involved, isn't there, really? I haven't really thought about a lot of this before. (All female FG1)

## 4 DISCUSSION

**Figure 4:**
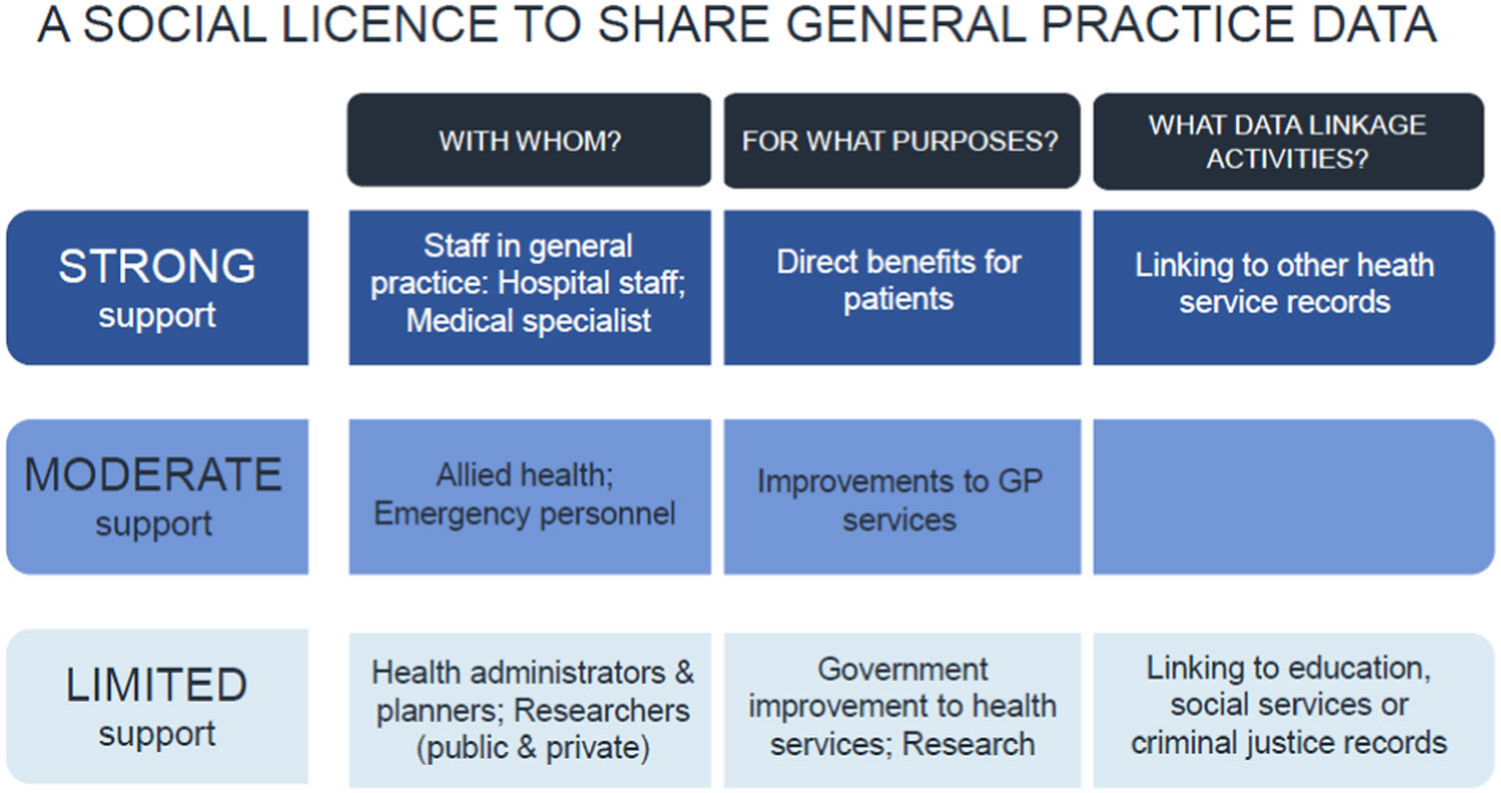
A social licence to share general practice data. GP, general practitioner.

This focus group study and survey weighted to be nationally representative provides preliminary evidence that there is a social licence in Australia to share general practice data, for the primary purpose of collection, that is, the provision of health care to individuals. The participants in this study strongly supported sharing general practice data with the clinicians responsible for their care, and where there were direct benefits for individual patients. There was also support for linking general practice data with those of other health service providers, provided that strong privacy protections were in place. Participants in the focus groups also talked positively about the potential for broader health and social benefits, with these views reflected, to some degree, in the survey participants' willingness to consider sharing health data for secondary purposes such as research and improvements in health services. (Figure 4 provides a pictorial summary of these findings.)

The extent to which this social licence extended to sharing general practice data beyond clinical care providers and activities related directly to clinical care was unclear. However, most survey participants did not support, or were unsure about, sharing general practice data with health administrators, planners and researchers (in both the public and private sectors) for research and health service planning. There was also little support amongst survey participants for linking general practice data to data in the education, social service and criminal justice systems. Again, here, a substantial minority of focus group and survey respondents were unsure or could not see how specific benefits would arise from sharing data.

There is a growing number of systematic, scoping and narrative reviews on community perspectives on sharing health data for secondary purposes, including for research, with which the findings in this study can be compared.^4^, ^6^, ^7^, ^8^, ^41^, ^42^, ^43^, ^44^, ^45^, ^46^ These reviews show that there is broad public support for sharing personal health data for secondary purposes, provided that a number of conditions are met:

1. the data must be used in the public interest;
2. privacy and security concerns must be addressed;
3. information must be communicated widely and transparently; and
4. the data must be deidentified and anonymised.

Views about consent and control over use of data are more variable but, even here, the reviews report that many participants are willing to accept models of consent with a lower level of individual control when the above conditions are met.^7^, ^43^, ^44^, ^45^

The reviews summarised above do not separate out community views about sharing administrative health data from views specifically about sharing general practice data. Those studies that do focus on the use of general practice data suggest that views about sharing general practice data may differ slightly from views about sharing data collected in other settings, with less support for sharing outside the clinical encounter and a greater preference for opt‐in consent.^15^, ^16^, ^17^, ^18^, ^19^ Our findings concur with these studies, and also suggest reasons why there may be less support for sharing general practice data. The focus groups and the qualitative comments in our survey indicate that at least some participants believed that the more personal nature of the GP–patient relationship created additional obligations to protect confidentiality that perhaps other data users in other health settings might not have.

How might we establish a broader social licence to share general practice data for secondary purposes, particularly one that can incorporate health service planning and research? As noted in the introduction, a social licence is granted where an enterprise meets certain standards of trustworthiness and legitimacy, in line with community expectations.^12^, ^47^ The findings in this paper provide guidance on these standards for a wider array of secondary uses of general practice data and the concerns and conditions that will need to be addressed in the process of developing a social licence. Addressing community concerns will not, in itself, be sufficient to generate a social licence. Generating social licence requires public engagement and consultation, the building of trust and the establishment of legitimacy over time. In the paragraphs that follow, we offer some starting points for building social licence for the secondary use of general practice data.

First, community members will need to be persuaded that there will be real benefits to the community from sharing their data, beyond the immediate benefits for patients that they can already see. This links to the foundation stones of social licence—trust and legitimacy—which both require that actions be guided by the common good and contribute to the well‐being of the community. There is good evidence from studies using deliberative methods^48^, ^49^, ^50^, ^51^ that community members can appreciate the value of data‐intensive research and such approaches could also be used to build confidence in the secondary use of general practice data.

Second, concerns about the potential for privacy breaches, discrimination and data misuse will need to be addressed. The participants in this study were concerned that their data might be used by GPs, researchers or insurance companies for their own personal profit. This links directly to trust and trustworthiness as the qualities of trustworthiness include competence to appropriately protect data and adequate consideration for the well‐being of participants and the community so as to avoid misuse of the data, including unjustified discrimination.

Many participants in this study wanted regulations and policies to control why, what, when and with whom data could be shared, and systems in place to keep data secure, avoid data breaches, ensure that individuals were not identified and limit access. For most participants, these controls were a condition of their support. Some of these controls are already in place in Australia, but they are not necessarily well understood by patients. For example, where data are reasonably identifiable, they are covered by privacy legislation. Where data are to be used for a new research project, that project is subject to approval by a human research ethics committee that requires that participants, and their data, are appropriately protected from both a privacy and security perspective and that the only individuals who have access to the data are those conducting research. In addition, researchers are required to ensure that individuals are not identifiable, without consent, in any publications that may result from the research. Raising awareness about existing regulatory and oversight mechanisms has the potential to contribute to both trust and legitimacy, particularly if the process is collaborative and reflective.

Third, the participants in this study wanted transparency surrounding the information collected from the general practice record. They wanted to know who had access to their information, what they used it for and for how long it would be kept. Although most participants in the survey were willing to trust that their GP would take appropriate care of their information, they were not necessarily ready to let their GP decide, without telling them, that their data could be shared for secondary purposes. There is good evidence that patients are not familiar with the extent to which information from general practice records is already being shared,^16^, ^17^, ^18^, ^34^, ^52^ suggesting a need for a substantial programme of dissemination and education, co‐designed with patients and publics. Transparency is a key element in establishing trust and will be key in seeking to establish the relevant social licence.

Fourth, nearly all participants wanted their names, addresses and dates of birth removed before data were shared. In the focus groups, the rationale for this was clear: most participants believed that this would ensure that their data were ‘anonymised’ or ‘deidentified’. However, removing names, addresses and dates of birth no longer means that individuals are necessarily anonymous or nonidentifiable.^53^, ^54^ In the focus group component of this study, only a small number of participants in this study understood this, and so shifting public views about sharing data away from a focus on data anonymisation as the vehicle for privacy protection and towards high standards in data oversight, security and accountability will be a key challenge.

Fifth, most participants wanted the opportunity to consent to data sharing, regardless of who the data would be shared with or for what purpose, although this condition was moderated to some extent in the focus groups by receiving information about the challenges with using opt‐in consent. Recent Australian studies on sharing data for health and medical research suggest that support for sharing data without consent is greater than we found in our focus groups and survey.^55^, ^56^, ^57^, ^58^ These differences may reflect the general practice context and the more personal nature of the GP–patient relationship. Whatever the reason, considerable public engagement will need to be undertaken to avoid a repeat of the UK *care.data* experience.

Finally, although community members may have specific concerns about sharing general practice data, the findings also suggest that GPs can play a central role as trusted agents who can build patients' confidence in the secondary use of general practice data. Although the provision of holistic, comprehensive and continuous care through general practice ideally positions GPs to fulfil this role, organisational constraints, lack of time and GPs' concerns about data sharing will need to be addressed if GPs are to do this.^59^, ^60^, ^61^, ^62^

## 5 LIMITATIONS

Both methods used in this study had limitations. First, because our participants were part of an online research panel and had chosen to participate after hearing about the research goals, they may be more interested in the topic area, including in issues related to data privacy and security.

Second, the online environment used for our focus groups presented a challenge; our moderator was only able to observe the participants' facial cues, and not their body language, which could have provided additional context for participants' responses. Although we tried to identify in advance and address technological issues, a few participants did experience problems with technology, which may have influenced the natural flow of the discussion. In addition, as is often the case for focus groups, we were aware that some participants might find it more difficult than others to voice their opinions, and that they could be influenced by the views of other participants or by what they thought we as researchers might want to hear. We used a range of strategies to encourage honest participation by providing written information before the focus groups to encourage participants to think about the topic in advance. We also included a number of individual and group activities during the focus groups and maintained a nonjudgemental and open environment.

Third, content validity of our survey was developed using a variety of items found in the literature overseen by experts in the field as our literature review did not identify a ‘gold standard’ tool to replicate.^63^

Finally, despite our best efforts to explain concepts of data collection, sharing and linkage, anonymity, privacy and security, we do not know the extent to which respondents in our online survey fully understood this complex topic. The challenges in explaining these concepts effectively in surveys and the variable understanding amongst community members have been noted as limitations in many other surveys on views about data sharing.^5^, ^22^, ^23^, ^27^ For example, within the confines of our short survey, we were unable to provide information about the impact of different consent models on data quality, bias and generalisability of findings, or to explain the role of trusted research environments. These are key topics to explore in future research, and deliberative methods would be useful to fully explore the views and concerns of an informed community.

## 6 CONCLUSION

Internationally and in Australia, the demands by researchers, planners and evaluators for access to general practice data are growing.^3^, ^64^ However, our findings suggest that there is substantial work still to be done in Australia to meet these demands, with current support to use general practice data outside of the clinical encounter probably extending only to quality assurance and, in some settings, to evaluation. Support for sharing for research and planning or with nonclinicians who are not directly involved with patient care is not strong. Securing legitimacy for sharing general practice data more broadly will require careful attention to patient and public concerns, including focusing on the factors that will sustain trust in general practice and GPs.

## AUTHOR CONTRIBUTIONS

**Annette J. Braunack‐Mayer**: Conceptualization; methodology; investigation; formal analysis; writing—original draft; writing—review & editing; visualization; supervision; funding acquisition. **Carolyn Adams**: Conceptualization; methodology; writing—review & editing; funding acquisition **Alberto Nettel‐Aguirre**: Methodology; software; data curation; investigation; formal analysis; resources writing—original draft; writing—review & editing; visualization. **Belinda Fabrianesi**: Methodology; investigation; writing—original draft; writing—review & editing; resources; visualization; project administration. **Lucy Carolan**: Methodology, investigation; writing—review & editing; resources; project administration. **Justin Beilby**: Conceptualization; writing—review & editing. **Felicity Flack**: Conceptualization; methodology; writing—review & editing.

## CONFLICT OF INTEREST STATEMENT

Dr Felicity Flack is employed by PHRN, who partially funded this research project. The remaining authors declare no conflict of interest.

## ETHICS STATEMENT

This study was approved by the University of Wollongong (UOW) Ethics Committee (Ethics number: 2021/343 and Ethics number: 2022/012). Focus group participants provided verbal consent before participating, while survey participants provided tacit consent before participating by indicating that they had reviewed an online participant information sheet.

## Supporting information

Supporting information.

Supporting information.

Supporting information.

Supporting information.

Supporting information.

Supporting information.

Supporting information.

## Data Availability

The data that support the findings of this study are available on request from the corresponding author. The data are not publicly available due to privacy or ethical restrictions.
