# Supplementary material for: Community views on the secondary use of general practice data: Findings from a mixed‐methods study
Source: Health Expect. 2024 Feb 15;27(1):e13984. doi: 10.1111/hex.13984 (PMC10869884; doi:10.1111/hex.13984)
Supplement: Supplementary file 1 — Supporting information. [file HEX-27-e13984-s003.docx]

## **Appendix 1: Case Studies**

## **Scenario 1:**

Maria is 65 years old. She has Type 2 Diabetes and Heart Disease. She and her husband have been attending the same general practice for thirty years. Today, she goes to see her GP to get repeat prescriptions for her diabetes and heart medications. The GP takes notes on the computer in Maria’s health record.

## **Scenario 2:**

Maria returns to see her GP four weeks later with an injured and badly infected toe. Her GP refers her to the local hospital for admission because she is worried Maria may lose her toe.

Maria has visited the hospital a number of times, including to see a heart specialist. In the Emergency Unit the doctor makes notes in Maria’s hospital record.

## **Scenario 3:**

Maria’s GP, along with all the other GPs in the practice and surrounding practices, has agreed to provide their patient records to contribute to a government funded program that aims to create a picture of all of the health care that people in Australia receive and the outcomes they experience. The names, addresses and dates of birth are removed from the individual patient records before they are shared.

At the moment, the program is particularly interested in patients with Type 2 Diabetes. Below are four examples of how information from people like Maria who have diabetes is shared.

1. The information about patients with diabetes (without their names, addresses and dates of birth) is shared with staff in a government department who compile a report for each GP taking part in the program. The GP receives a report comparing her patients diabetes outcomes are doing compared the patients of all other GPs in the state.
2. The information about patients with diabetes (without their names, addresses and dates of birth) is shared with staff in a government department. Government staff use information to prepare a report on planning where to put new diabetes services and what sorts of services to provide.
3. The information about patients with diabetes (without their names, addresses and dates of birth) is shared with researchers in a university. Researchers use information to see if they can predict what might make someone’s diabetes get better or worse.

## **Scenario 4:**

A special coordinating unit brings together some of the general practice data about each patient with other health information about the same patient from other general practices, emergency departments, public and private hospitals, other health professionals such as physiotherapists and occupational therapists, and ambulance records. Remember that there are no names, addresses, and dates of birth included when this information is shared.

1) A researcher looking at outcomes for people with Type 2 diabetes and heart disease would like to analyse data covering all general practice visits by people with diabetes.

**Q: Is it okay to link information about a person from one general practice with their information from another practice?**

2) Researchers want to combine data from hospital records, general practice records and other health professional records, to answer a wide range of questions about diabetes and heart disease in the community.

**Q: Is it okay to link information about a person from all of these records (general practice, hospital, other health professionals and ambulance)?**
