# Supplementary material for: Community views on the secondary use of general practice data: Findings from a mixed‐methods study
Source: Health Expect. 2024 Feb 15;27(1):e13984. doi: 10.1111/hex.13984 (PMC10869884; doi:10.1111/hex.13984)
Supplement: Supplementary file 2 — Supporting information. [file HEX-27-e13984-s001.docx]

# Appendix 2: COREQ

| Research team and reflexivity | |
| --- | --- |
| *Personal characteristics* | |
| Interviewer/facilitator | Lead author ABM facilitated all focus groups |
| Credentials | PhD (Bioethics) BMedSci |
| Occupation | Head of School of Health and Society, University of Wollongong |
| Gender | Female moderator x 1, female research assistants x 2, 2 x female observers, 2 x male observers |
| Experience and training | Professor Braunack-Mayer is a bioethicist, with extensive experience in the use of qualitative research methods to explore public health issues, including data linkage, obesity, immunization and pandemics. Previous work includes research on the place of privacy and confidentiality in medicine and public health, the role of ethics in the education of medical students, and the relationship between ethics and health technology assessment. |
| *Relationships with participants* | |
| Relationship established | No relationship prior to the focus groups |
| Participant knowledge of the interviewer | Participants received a Participant Information Sheet (PIS) which described the moderator’s role and provided contact details e.g., ‘lead Chief Investigator Professor Annette-Braunack-Mayer at abmayer@uow.edu.au’ |
| Interview characteristics | Participants were provided with a brief introduction in the PI: ‘We are undertaking focus groups to investigate community knowledge, attitudes and values with respect to use of people’s general practice data being used for research purposes, as well as through linking general practice data to other data sources.’ |
| Domain 2: Study design | |
| *Theoretical framework* | |
| Methodological orientation and theory | Focus groups have their methodological origins in marketing and social research. They are designed to encourage discussion in a supportive environment and non-confrontational way to understand people’s perspectives, attitudes and values. ^1, 2^ In this project, the theoretical framework for the focus groups is empirical bioethics, focusing on how the participants’ knowledge, perspectives and values interact. |
| *Participant selection* | |
| Sampling | Sampling for focus groups follows a qualitative (purposive) rather than a quantitative (representational) sampling strategy. Our aim was to constitute broadly inclusive groups, with a range of ages (18 years+), genders, SES and cultural backgrounds, from a range of geographic areas nationally, including urban, regional and rural), in order to recruit as diverse a sample as possible. |
| Method of approach | We employed an experienced Australian market research company, McNair yellowSquares ^3^ to recruit a sample of 24 participants from their opt-in online panel. McNair yellowSquares were asked to secure a diverse sample with respect to gender, age, geographic residential location (rural, regional and urban), national spread, educational qualifications, employment and cultural background. Only panel members 18 years and older were invited. We excluded potential participants with current or previous experience(s) employed in a general practice setting to limit the impact of firsthand knowledge of data sharing practices in general practice. Using a screening survey, participants were purposively selected from MacNair yellowSquare’s panel to meet these requirements. |
| Sample size | 24 participants were recruited (22 participants took part in the focus groups) |
| Non-participation | 2 participants failed to attend on the day despite recording verbal consent to participate in the week before hand. |
| *Setting* | |
| Setting of data collection | On-line Focus group via Zoom |
| Presence of non-participants | The moderator (ABM) and two members of the research team (BF & LC) plus one observer per group |
| Description of samples | Focus Group 1 - All female group (25 years+),  Focus Group 2 - All male group (25 years +),  Focus Group 3 - Mixed group all ages (25 years +),  Focus Group 4 - Young persons’ group (18 – 25 years), |
| *Data collection* | |
| Interview guide | A moderator guide was developed for the research team and moderator use. A participant information pack which included the case studies to be discussed during the focus groups was posted out one week ahead of the focus groups (see Appendix 1)  The focus group was pilot tested and minor amendments to the discussion/ moderator guide made after the pilot. |
| Repeat interviews | Four focus groups were held in total, within a 2-week period. |
| Audio/visual recording | All focus groups were audio recorded and transcribed. |
| Field notes | Notes were taken during the focus groups by a research team member. |
| Duration | Focus groups were 90 minutes in duration |
| Data Saturation | Data saturation was discussed, and determined to have been achieved by the fourth focus group |
| Transcripts returned | Transcripts were not returned to participants for comment and/ or correction |
| Domain 3: analysis and findings | |
| *Data analysis* |  |
| Number of data coders | Two members of the research team (ABM & LC) |
| Description of the coding tree | The coding tree focused on concepts related to: awareness and understanding of the content of GP and hospital records; support for sharing and linkage (including the perceived benefits); concerns about sharing and linkage; conditions and controls on sharing and linkage |
| Derivation of themes | Themes were both proposed in advance and also derived from the data |
| Software | Microsoft Word, Excel and NVivo were used to manage the data |
| Participant checking | Participants did not provide feedback |
| *Reporting* | |
| Quotations presented | Quotations from focus group participants are presented to illustrate findings and are identified with a focus groups and participant number |
| Data and findings consistent | There was consistency between the data presented in the literature search and the focus group findings. |
| Clarity of major themes | Major themes are clearly presented in the findings. |
| Clarity of minor themes | Minor themes are clearly presented in the findings. |

**References:**

1. Morgan D. *Focus groups as qualitative research*. 2nd ed. Sage Publications; 1997.

2. Kitzinger J. Qualitative Research: Introducing focus groups. *BMJ*. 1995;311(7000):299-302. doi:10.1136/bmj.311.7000.299

3. McNair yellowSquares. McNair yellowSquares. Accessed 9th February, 2022. <https://mcnair.com.au/>
