# Supplementary material for: Community views on the secondary use of general practice data: Findings from a mixed‐methods study
Source: Health Expect. 2024 Feb 15;27(1):e13984. doi: 10.1111/hex.13984 (PMC10869884; doi:10.1111/hex.13984)
Supplement: Supplementary file 3 — Supporting information. [file HEX-27-e13984-s006.docx]

**Appendix 3: Online Survey Instrument**

**PAGE 1**

**Eligibility Criteria**

We are asking the following three questions so that we can check how closely our sample matches the whole population of Australia

**1. With which gender do you most identify?** One response only

|  | Female |  |  | Non-binary |
| --- | --- | --- | --- | --- |
|  | Male |  |  | I prefer to describe: __________(free text 50 characters) |
|  | I prefer not to say |  |  |  |

**2. How old are you?** Insert numerals only. Please program for responses 17 and under to be immediately exited from the survey.

__________ in years

**3. Where do you currently live?** One response only

|  | Greater Sydney |
| --- | --- |
|  | Rest of NSW |
|  | Greater Melbourne |
|  | Rest of VIC |
|  | Greater Brisbane |
|  | Rest of QLD |
|  | Adelaide |
|  | Rest of SA |
|  | Perth |
|  | Rest of WA |
|  | TAS |
|  | Northern Territory |
|  | ACT |

**PAGE 2**

**Participant Information Sheet**

Please read the Participant Information Sheet below:

(Participant information sheet will appear on this page within the survey)

|  | Please tick the box to indicate you have read and understood the Participant Information Sheet Participant to indicate by ticking box |
| --- | --- |

If you would like to view and/or download and/or print the Participant Information Sheet please go here. This link will send participant to a PDF version of Participant Information Sheet.

**PAGE 3**

**Section A: Patient information in general practice**

**Please watch this video before answering question 4** link to video 1 (mandatory – able to be replayed)

**V**i**deo 1 text:**

In Australia, general practitioners (GPs) are the first and primary source of health care for most people.

Information about you is recorded in your general practice record, mostly in computers.

The record may include your symptoms, what the GP finds when they examine you, your blood test results, diagnoses, medications and other treatments, and communications with other health professionals.

The record may also include non-medical information, such as aspects of your personal life, work life, and/or family situation.

GPs sometimes share the information in patient records with other people and organisations.

Everyone who handles your information is covered by privacy laws or privacy principles as well as other legal obligations to protect the information in your record.

Information in your general practice record can only be shared for specific purposes.

In answering the first set of questions, please assume that the GP and everyone your information is shared with will abide by all relevant laws and will do everything they can to minimise risks to your privacy.

However, it is still possible that you may be identified from some of the information shared about you.

We are interested in understanding who you think your general practice information is being shared with and whether you agree with this.

In answering these questions, please assume that the GP and everyone your information is shared with will abide by all relevant laws and will do everything they can to minimise risks to your privacy. However, it is still possible that you may be identified from some of the information shared about you.

**4. Do you think information from your general practice record is being shared with the following people?**

Answer all items. One response per row.

|  | Yes | No | I do not know |
| --- | --- | --- | --- |
| (a) Me |  |  |  |
| (b) My immediate family members |  |  |  |
| (c) Other staff in the general practice, e.g., other GPs, practice nurse, |  |  |  |
| (d) Hospital health professionals e.g., doctors or nurses |  |  |  |
| (e) Emergency personnel e.g., ambulance staff |  |  |  |
| (f) Other medical specialists, e.g., surgeon, cardiologist |  |  |  |
| (g) Allied health professionals, e.g., pharmacist or psychologist |  |  |  |
| (h) Health administrators and planners in government |  |  |  |
| (i) Researchers in a university |  |  |  |
| (j) Researchers in a government department |  |  |  |
| (k) Health researchers in private industry, e.g., a pharmaceutical company |  |  |  |

**PAGE 4**

**Please click here if you would like to watch the video 1 again** link to video 1 (not mandatory – able to be replayed)

**5. To what extent do you agree with information from your general practice record being shared with the following people?**

Answer all items. One response per row.

|  | Strongly agree | Agree | Neither agree nor disagree | Disagree | Strongly disagree |
| --- | --- | --- | --- | --- | --- |
| (a) Me |  |  |  |  |  |
| (b) My immediate family members |  |  |  |  |  |
| (c) Other staff in the general practice, e.g., other GPs, practice nurse, |  |  |  |  |  |
| (d) Hospital health professionals e.g., doctors or nurses |  |  |  |  |  |
| (e) Emergency personnel e.g., ambulance staff |  |  |  |  |  |
| (f) Other medical specialists, e.g., surgeon, cardiologist |  |  |  |  |  |
| (g) Allied health professionals, e.g., pharmacist or psychologist |  |  |  |  |  |
| (h) Health administrators and planners in government |  |  |  |  |  |
| (i) Researchers in a university |  |  |  |  |  |
| (j) Researchers in a government department |  |  |  |  |  |
| (k) Health researchers in private industry, e.g., a pharmaceutical company |  |  |  |  |  |

**PAGE 5**

**Section B: For what reasons can general practice information be shared?**

**Please click here if you would like to watch the video 1 again** link to video 1 (not mandatory – able to be replayed).

There are a number of reasons why GPs share the information in your patient record with other people.

We are interested to know what you think are the reasons for your general practice information being shared and whether you agree with this.

In answering these questions, please assume that the GP and everyone your information is shared with will abide by all relevant laws and will do everything they can to minimise risks to your privacy. However, it is still possible that you may be identified from some of the information shared about you.

**6. Do you think information from your general practice record is being shared for the following reasons?**

Answer all items. One response per row.

|  | Yes | No | I do not know |
| --- | --- | --- | --- |
| (a) To directly support my personal health care |  |  |  |
| (b)For my GP to improve the health services they provide |  |  |  |
| (c) For the government to improve health services generally |  |  |  |
| (d) For research in universities, hospitals or publicly funded research organisations |  |  |  |

**PAGE 6**

**Please click here if you would like to watch the video 1 again** link to video 1 (not mandatory, able to be replayed)

**7. To what extent do you agree with information from your general practice record being shared for the following reasons?**

Answer all items. One response per row.

|  | Strongly agree | Agree | Neither agree nor disagree | Disagree | Strongly disagree |
| --- | --- | --- | --- | --- | --- |
| (a) To directly support my personal health care |  |  |  |  |  |
| (b)For my GP to improve the health services they provide |  |  |  |  |  |
| (c)For the government to improve health services generally |  |  |  |  |  |
| (d) For research in universities, hospitals or publicly funded research organisations |  |  |  |  |  |

**PAGE 7**

**Section C: Sharing and linking general practice information for research**

**Please watch this video before answering question 8** Link to video 2 (mandatory, able to be replayed)

**Video 2**

We would like to know more about your views on sharing information from your general practice record specifically for research.

Researchers can do more valuable research if they can bring together information, not just from your GP, but also from other places such as hospital, education, and social services records.

When your general practice information is used for research, there are special safeguards.

A Human Research Ethics Committee must consider each research project to ensure that the researchers will protect the privacy and security of your general practice information by,

for example, not sharing the information with anyone outside the research team and

not publishing anything that would identify individual patients.

To answer the questions which follow, we would like you to assume that the researchers will not have your name and address. However, it is still possible that the researchers may be able to work out who you are from information provided about you.

We would like to know what you think about information from your general practice record being linked with other information about you from other sources for specific research purposes. Please remember that the researchers will not have your name or address. However, it is still possible that the researchers may be able to work out who you are from information provided about you.

**8. To what extent do you agree with information from your general practice record being linked with information about you from the following sources for specific research purposes?**

Answer all items. One response per row.

|  | Strongly agree | Agree | Neither agree nor disagree | Disagree | Strongly disagree |
| --- | --- | --- | --- | --- | --- |
| (a) Other health service records, e.g., hospital and ambulance records |  |  |  |  |  |
| (b) Education department records |  |  |  |  |  |
| (c) Social service records, e.g., disability, housing and childcare services |  |  |  |  |  |
| (d) Criminal justice records |  |  |  |  |  |

**PAGE 8**

**SECTION D: Trust in GP and general practice records**

**9. To what extent do you agree with the following statements about your GP and sharing information from your general practice record?**

Please remember, the GP and everyone your information is shared with will abide by all relevant laws and will do everything they can to minimise risks to your privacy. However, it is still possible that you may be identified from some of the information shared about you.

Answer all items. One response per row.

|  | Strongly agree | Agree | Neither agree nor disagree | Disagree | Strongly disagree |
| --- | --- | --- | --- | --- | --- |
| (a) I am confident that my GP will take care of the information in my general practice record appropriately |  |  |  |  |  |
| (b) There are some aspects of the information in my general practice record I would rather my GP did not share with anyone else |  |  |  |  |  |
| (c) I would want to know when the information in my general practice record is being shared |  |  |  |  |  |
| (d) I would want to know who the information in my general practice record is being shared with |  |  |  |  |  |
| (e)I would want to know why the information in my general practice record is being shared |  |  |  |  |  |
| (f) I would be willing to let my GP decide who can see information from my health record without informing me |  |  |  |  |  |
| (g) My name, address and date of birth must be removed before my general practice information is shared |  |  |  |  |  |

**PAGE 9**

**10. If you have anything else to tell us about your views on using information from your general practice records, please comment below?**

|  | No |
| --- | --- |
|  | Yes (please respond below) |

Open text 2000 characters

|  |
| --- |

**PAGE 10**

**Demographics**

**11. In general, how would you rate your health?**

One response only

|  | My health is poor |  |
| --- | --- | --- |
|  | My health is fair |  |
|  | My health is good |  |
|  | My health is very good |  |
|  | My health is excellent |  |

**12. About your health status:**

Answer all items. One response per row.

|  | Yes | No | I am not sure/I do not know |
| --- | --- | --- | --- |
| (a) I have a chronic health condition |  |  |  |
| (b) I care for someone with a chronic health condition |  |  |  |
| (c) I take prescribed medication(s) |  |  |  |
| (d) I have a My Health Record electronic health record |  |  |  |

**13. In what type of setting did you most often see a GP in the last year,** **including face-to-face, online or by telephone?**

One response only

|  | General practice |
| --- | --- |
|  | Community centre, e.g., Women’s Health Centre, Aboriginal Medical Service |
|  | Residential or nursing care home |
|  | Hospital or rehabilitation |
|  | Other (please specify): |

**PAGE 11**

**14. How many times did you see a GP in the last year, including face-to-face, online or by telephone?**

One response only

|  | I did not see a GP in the last year |
| --- | --- |
|  | 1-3 times in the year |
|  | 4-12 times in the year |
|  | 13-52 times in the year |
|  | More than once a week |

**15. Which best describes the highest educational qualification you have obtained?**

One response only

|  | No formal qualifications |
| --- | --- |
|  | Year 10 or school certificate |
|  | Year 12 or leaving certificate |
|  | Trade/apprenticeship |
|  | Other TAFE/Certificate |
|  | University degree/Higher degree |
|  | I prefer not to answer/I am not sure |

**16. What best describes your current employment status?**

One response only

|  | Full time employed |  | Student/Training |
| --- | --- | --- | --- |
|  | Part-time employed |  | Retired |
|  | Unemployed |  | Unable to work (e.g., disability/Work Cover) |
|  | Home duties |  | I prefer not to answer/I am not sure |

**PAGE 12**

**17. What is your household’s combined income?**

One response only

|  | $1 - $19,999 per year ($1 - $379 per week) |
| --- | --- |
|  | $20,000 - $39,999 per year ($380 - $769 per week) |
|  | $40,000 - $59,999 per year ($770 - $1149 per week) |
|  | $60,000 - $99,999 per year ($1150 - $1919 per week) |
|  | $100,000 - $124,999 per year ($ 1920 - $2399 per week) |
|  | $125,000 - $149,999 per year ($2,400 - $2879 per week) |
|  | $150,000 - $199,999 per year ($2880 - $3839 per week) |
|  | $200,000 or more per year ($3840 or more per week) |
|  | I prefer not to answer/I am not sure |

**1****8. Have you worked, or do you currently work in the health industry and/or in health services or research?**

Tick all that apply (maximum 3 answers)

|  | Yes, in a general practice |
| --- | --- |
|  | Yes, in the health industry (other than general practice) |
|  | Yes, in conducting research (this does not include being part of a research panel) |
|  | No |
|  | I am not sure |
|  | I prefer not to answer |

**Thankyou for completing our survey**

**If you would like to view and/or download and/or print the Participant Information Sheet please go here.** This link will send participant to a PDF version of Participant Information Sheet.
