# Supplementary material for: Community views on the secondary use of general practice data: Findings from a mixed‐methods study
Source: Health Expect. 2024 Feb 15;27(1):e13984. doi: 10.1111/hex.13984 (PMC10869884; doi:10.1111/hex.13984)
Supplement: Supplementary file 4 — Supporting information. [file HEX-27-e13984-s002.docx]

**Appendix 4 SURGE: Data abstraction quality criteria**

| Category | Item |
| --- | --- |
| Title and abstract | |
| Is the design of the study stated in the title and/or abstract? | Yes. The design of the study, a ‘cross-sectional survey’, is stated in the title of the manuscript. |
| Is there an explanation of why the research is necessary, placing the study in context of previous work in relevant fields? | Yes. In the manuscript an explanation of why the research is necessary is stated in the introduction. |
| Is the purpose or aim of the paper explained? | Yes. In the manuscript the aim of the paper is explained in the introduction, “we explored what the Australian public knows about the secondary use of the information in their general practice records”. |
| Method | |
| **Research tools** | |
| Is the questionnaire described? | Yes. We developed our online survey by initially reviewing the existing literature on public understandings of, and attitudes towards, using the information captured in general practice records for secondary purposes. We examined peer reviewed and grey literature to identify quantitative and qualitative tools measuring public views, with a focus on instruments examining sharing for purposes beyond patient care or quality assurance. We combined questions from pre-existing tools with new questions and insights from the literature ^1-11^, overseen by the research team’s knowledge and expertise. We also incorporated findings from the focus groups, particularly concerning participants’ knowledge of when, why and with whom general practice data were shared.  Two videos were included in the survey to enhance participants’ understanding of data sharing in general practice. ^12^ The first introduced the survey and explained: the types of information captured in general practice records; how information in general practice records may be shared with other people or organisations and for what purposes; privacy principles and privacy laws; and the very small risk of identifiability. The second video explained how information from general practice records could be linked with other records, the role of a Human Research Ethics Committee, privacy protections and again mentioned the small risk of identifiability. Introductory Video 1 can been viewed here: (<http://ro.uow.edu.au/asshpapers/461>). Video 2 can be viewed here: (http://ro.uow.edu.au/asshpapers/462)  The final survey (including videos) took approximately 10 minutes to complete.  The 18-question instrument examined community knowledge and views across four domains (See Appendix 3 for our survey instrument):  1. Knowledge of how information held in general practice records was shared with specific people or organisations and for specific purposes  2. Views about whether information held in general practice records should be shared with specific people or organisations and for specific purposes  3. Views on linking general practice records with other data sources for research purposes  4. Views about the trustworthiness of GPs, identifiability and transparency with respect to sharing information in general practice records  We also collected sociodemographic characteristics, health status and GP attendance for participants. A single open-ended question at the end of the survey invited additional comments. |
| If an existing tool was used, are its psychometric properties presented? | Not applicable. We did not use an existing tool. |
| If an existing tool was used, are references to the original work provided? | Not applicable. We did not use an existing tool. |
| If a new tool was used, are the procedures used to develop and pre-test provided? | Yes. We piloted a hardcopy version of the survey with a convenience sample of the general population (n=9) aged 34 years and over. ^13^ Pilot participants were diverse with respect to age, gender, education and ethnicity. The survey was then amended based on participants’ feedback before being programmed by the research company, McNair yellowSquares, on the Web Survey Creator survey platform. The research team checked the survey for usability and technical functionality before launching. |
| If a new tool was used, have its reliability and validity been reported? | No. As this tool did not aim to evaluate or measure a construct such as “knowledge” (for example), as such no strict psychometrics on reliability and validity have been performed. |
| Is a description of the scoping procedures provided? | Not applicable |
| **Sample selection** | |
| Is there a description of the survey population and the sample frame used to identify this population? | Yes. Members of the Australian public may opt in to sign up to be part of a McNair yellowSquares ^14^ online research panel. McNair yellowSquares aimed to recruit an opt-in sample of 2,500 Australian participants drawn from its online panel, selected to be nationally representative by age, gender and location. While potentially less ideal than probability sampling, this methodology had the practical advantage of ease of implementation and was considered appropriate for this exploratory study. ^15^ |
| Do the authors provide a description of how representative the sample is of the underlying population? | Yes. To support population inference, we analysed the survey data using post stratification using gender, age, place of residence, and highest educational attainment weights. We used the 2016 Australian Bureau of Statistics census data ^16^ to obtain the Australian population characteristics of gender, age, state and education and calculate the survey weights based on the realised sample characteristics after combining categories with small sample counts. The rake method ^17^ was used due to the fact that not all possible crossings of all possible levels of the variables chosen were observed in the data. Hence the marginal tables of Sex and Age, Sex and Residence, Age and Highest education attained, Sex and Highest Education attained, were all used for the Rake method. All results in this paper except for participant demographics are obtained using such weights.  **Analysis: A note on Australian Bureau of Statistics weighting**  Australian Bureau of Statistics only has in their most recent 2016 Census available for use with the table builder Sex as Male and Female, hence the 12 participants in our data that did not identify directly with either of these, had to be excluded as otherwise they would not be receiving a weight. Weighting was planned taking into account of the following variables:   \| Values considered \| ABS equivalent \| \| --- \| --- \| \| Age in groups \| \| \| 18-24 years, 25-29 years, 30-34 years, 35-39 years, 40-44 years, 45-49 years, 50-54 years, 55-59 years, 60-64 years, 65-69 years, 70-74 years, and 75 or more  Of Note, our data only had a maximum observed age of 88, and so “75 or more” is, in reality, 75-88. Otherwise, we would be falsely giving much more weight to that group \| Same \| \| Gender \| \| \| Female, Male \| Same \| \| Place of residence \| \| \| Sydney, NSW other than Sydney, Melbourne, VIC other than Melbourne, Brisbane, QLD other than Brisbane, Perth, WA other than Perth, Adelaide, SA other than Adelaide, NT, TAS, ACT. \| Pretty much the same except for labelling e.g., “Greater Sydney”, “NSW not Greater Sydney”, etc \| \| Highest attained education \| \| \| Based on survey \| Based on Australian Standard Classification of Education (ASCED), 2001 \| \| "No formal, not described” formed from: "No formal qualifications”, “I prefer not to answer/I am not sure") \| 622- Year 9  623- Year 8  624- Year 7 (excluding SA)  All 7, 8 and 9 levels \| \| "Year 10 or school certificate" \| 621 -year 10  613- year 11  612- Bridging and Enabling Course at Senior Secondary Level \| \| "Year 12 or leaving certificate" \| 611- Year 12 \| \| "TAFE/Certificate/Trade" formed from: "Trade/apprenticeship", "Other TAFE/Certificate" \| All 4 and 5 \| \| "University degree/Higher degree" \| All 1, 2 and 3 \| |
| Is a sample size calculation or rationale/justification for the sample size presented? | Yes. The rationale for the sampling approach to recruit 2,500 participants was based on our previous research using a national online survey. ^18^ As our questions resulted in categorical variable measures for each question, we knew we would be estimating proportions. Before any weighting, and for a crude estimate of proportions, the sample of 2500 was appropriate as it would allow for a worst-case scenario Margin of Error of 2% in constructing 95% confidence intervals. |
| **Survey administration** | |
| Mode of administration? | Yes. McNair yellowSquares emailed participants of their online panel an invitation to participate in the closed online survey via a unique one-time use link. Once the survey was completed, the link was disabled to prevent duplicates and the panel was regularly checked for duplication with various data points.  Upon completing the introductory section to establish the quotas, participants were directed to the Participant Information Sheet which described the researchers, purpose of the study, risks and benefits, time involved to complete and data protection and storage. Participants were asked to indicate that they understood the information sheet; on assenting to this, they were directed to the first page of the survey. All quantitative questions were mandatory, and some items provided an ‘I do not know’ or ‘I prefer not to answer’ response option. Participants were not able to view their responses by moving backwards. There was no randomisation of items, and all responses were captured on the McNair yellowSquares Web Survey Creator survey platform. |
| Do the authors provide information on the type of contact and how many attempts were made to contact subjects (i.e., prenotification by letter or telephone, reminder postcard, duplicate questionnaire with reminder)? | Yes. The survey was not advertised in any manner. Up to two reminder emails were sent over the three-week period during which the survey was open (18^th^ March to 7^th^ April 2022). |
| Do the authors report whether incentives were provided (financial or other)? | Yes. Participation was voluntary and participants received a small reward on completion of all items in the survey. |
| Is there a description of who approached potential participants (e.g., identification of who signed the covering letter)? | Yes. McNair yellowSquares approached individuals who had signed up to be part of a McNair yellowSquares online research panel. |
| Analysis | |
| Is the method of data analysis described? | Yes. We used R Project for Statistical Computing ^19^ to analyse the data. Only completed survey data were analysed. First, we provided a descriptive summary of the survey outcomes by showing a frequency table with relative frequencies for each question of interest. |
| Do the authors provide methods for analysis of nonresponse error? | Not applicable. The dataset only contains responses from participants who completed the full survey. |
| Is the method for calculating response rate provided? | Not applicable. A response rate cannot be calculated. The online survey was emailed to 24, 787 members of the McNair yellowSquares online research panel. 3,785 responded within 21 days. The survey was closed once the quotas for age, gender and location were met. |
| Are definitions provided for complete versus partial completions? | Not applicable. All quantitative questions were mandatory, and some items provided an ‘I do not know’ or ‘I prefer not to answer’ response option. The dataset contains responses from participants who completed the full survey. |
| Are the methods for handling item missing data provided? | Not applicable. All quantitative questions were mandatory, and some items provided an ‘I do not know’ or ‘I prefer not to answer’ response option. The dataset contains responses from participants who completed the full survey. |
| Results | |
| Is the response rate reported? | Yes. In total, 2,604 participants were included in our survey sample. |
| Are all respondents accounted for? | Yes. The dataset only contains responses from participants who completed the full survey. |
| Is information given on how nonrespondents differ from respondents? | No. The concept of non-responders is less relevant for surveys conducted with panels. The online survey was open to all members of the McNair yellowSquares online research panel. The survey was closed once the quotas for age, gender and location were met. Demographic information about the non-respondents and those who commenced but did not complete the survey was not captured. |
| Are the results clearly presented? | Yes. In the manuscript the results are presented objectively in tables and figures. |
| Do the results address the objective(s)? | Yes. In the manuscript we aligned our results to the study aims. |
| Discussion | |
| Are the results summarized with reference to the study objectives? | Yes. In the manuscript the results are summarised with reference to the study objectives. |
| Are the strengths of the study stated? | Yes. In the manuscript the strengths of the study are summarised in the discussion and abstract. |
| Are the limitations of the study (taking into account potential sources of bias or imprecision) stated? | Yes. There are number of limitations in this study related to the use of an online panel. The survey respondents were members of the Australian public who had expressed interest and willingness to participate in research and may be considered more likely to be supportive of research, or at least more interested, than the general public. Participating in online research activities may also indicate our respondents have a reasonable level of confidence in using information technology and the internet, although what this may mean for their attitudes to sharing information from their general practice record is unclear.  Content validity of our survey was developed using a variety of items found in the literature overseen by experts in the field. A full formal validation exercise of our instrument was not undertaken, and our literature review did not identify a ‘gold standard’ tool to replicate. ^20^  In addition, despite our best efforts to explain concepts of data collection, sharing and linkage, and anonymity, we do not know the extent respondents in our online survey fully understood this complex topic. The challenges in explaining these concepts effectively in surveys and the variable understanding amongst community members have been noted as limitations in many other surveys on views about data sharing. ^1, 6, 18, 21^ Additional deliberative methods would be useful to fully explore the views and concerns of an informed community. ^13^ |
| Is there explicit discussion of the generalizability (external validity) of the results? | Yes. Our survey is limited to the Australian healthcare landscape and did not include the views of Australians under 18 years old. |
| Ethical quality indicators | |
| Is study funding reported? | Yes. This project received funding support from the Digital Health CRC Limited (DHCRC) and Population Health Research Network. The Digital Health CRC Limited is funded under the Commonwealth's Cooperative Research Centres (CRC) Program. The Population Health Research Network is a capability of the Australian Government National Collaborative Research Infrastructure Strategy. |
| Research Ethics Board (REB) review reported? | Yes. This study was approved by the University of Wollongong (UOW) Ethics Committee (Ethics number: 2022/012). |
| Reporting of subject consent procedures? | Yes. All participants provided tacit consent before participating by indicating that they had reviewed an online participant information sheet. |

**References:**

1. Buckley B, Murphy A, MacFarlane A. Public attitudes to the use in research of personal health information from general practitioners' records: a survey of the Irish general public. *Journal of Medical Ethics*. Jan 2011

2016-04-15 2011;37(1):50. doi:<http://dx.doi.org/10.1136/jme.2010.037903>

2. Cherif E, Bezaz N, Mzoughi M. Do personal health concerns and trust in healthcare providers mitigate privacy concerns? Effects on patients’ intention to share personal health data on electronic health records. *Social Science & Medicine*. 2021/08/01/ 2021;283:114146. doi:<https://doi.org/10.1016/j.socscimed.2021.114146>

3. Clerkin P, Buckley B, Murphy A, MacFarlane A. Patients’ views about the use of their personal information from general practice medical records in health research: A qualitative study in Ireland. *Family Practice*. 2012;30(1):105-112. doi:10.1093/fampra/cms036

4. Curved Thinking. *Understanding public expectations of the use of health and care data*. 2019. <https://understandingpatientdata.org.uk/sites/default/files/2019-07/Understanding%20public%20expectations%20of%20the%20use%20of%20health%20and%20care%20data.pdf>

5. Ghafur S, Van Dael J, Leis M, Darzi A, Sheikh A. Public perceptions on data sharing: Key insights from the UK and the USA. *The Lancet Digital Health*. 2020/09/01/ 2020;2(9):e444-e446. doi:<https://doi.org/10.1016/S2589-7500(20)30161-8>

6. O’Brien E, Rodriguez A, Kum H, et al. Patient perspectives on the linkage of health data for research: Insights from an online patient community questionnaire. *International Journal of Medical Informatics*. 2019/07/01/ 2019;127:9-17. doi:<https://doi.org/10.1016/j.ijmedinf.2019.04.003>

7. Perera G, Holbrook A, Thabane L, Foster G, Willison DJ. Views on health information sharing and privacy from primary care practices using electronic medical records. *International Journal of Medical Informatics*. 2011/02/01/ 2011;80(2):94-101. doi:<https://doi.org/10.1016/j.ijmedinf.2010.11.005>

8. Powell J, Fitton R, Fitton C. Sharing electronic health records: the patient view. *Informatics in Primary Care*. 2006;14(1):55-57. doi:<http://dx.doi.org/10.14236/jhi.v14i1.614>

9. Sanyer O, Butler J, Fortenberry K, Webb-Allen T, Ose D. Information sharing via electronic health records in team-based care: The patient perspective. *Family Practice*. 2021;38(4):468-472. doi:10.1093/fampra/cmaa145

10. Stone M, Redsell S, Ling J, Hay A. Sharing patient data: Competing demands of privacy, trust and research in primary care. *British Journal of General Practice*. Oct 2005;55(519):783-9.

11. Whiddett R, Hunter I, Engelbrecht J, Handy J. Patients’ attitudes towards sharing their health information. *International Journal of Medical Informatics*. 2006/07/01/ 2006;75(7):530-541. doi:<https://doi.org/10.1016/j.ijmedinf.2005.08.009>

12. Riggs E, Azzariti D, Niehaus A, et al. Development of a consent resource for genomic data sharing in the clinical setting. *Genetics in Medicine*. Jan 2019;21(1):81-88. doi:10.1038/s41436-018-0017-5

13. Kelly K, Clark B, Brown V, Sitzia J. Good practice in the conduct and reporting of survey research. *International Journal for Quality in Health Care*. 2003;15(3):261-266. doi:10.1093/intqhc/mzg031

14. McNair yellowSquares. McNair yellowSquares. Accessed 9th February, 2022. <https://mcnair.com.au/>

15. Pennay D, Neiger D, Lavrakas P, Borg K. *The Online Panels Benchmarking Study: A total survey error comparison of findings from probability-based surveys and non-probability online panel surveys in Australia*. 2018. <https://csrm.cass.anu.edu.au/research/publications/online-panels-benchmarking-study-total-survey-error-comparison-findings>

16. Austrlalian Government. Australian Bureau of Statistics: Statistics. Accessed 14th March, 2022. <https://www.abs.gov.au/statistics>

17. Lumley T. Analysis of Complex Survey Samples. *Journal of Statistical Software*. 2004;9(8)doi:10.18637/jss.v009.i08

18. Braunack-Mayer A, Fabrianesi B, Street J, et al. Sharing government health data with the private sector: Community attitudes survey. *Journal of Medical Internet Research*. Oct 2021

2021-11-01 2021;doi:<http://dx.doi.org/10.2196/24200>

19. The R Foundation. The R Project for Statistical Computing. Accessed 4th February 2022, <https://www.r-project.org/>

20. Boateng G, Neilands T, Frongillo A, Melgar-Quiñonez H, Young S. Best practices for developing and validating scales for health, social, and behavioral research: A primer. *Front Public Health*. 2018;6:149-149. doi:10.3389/fpubh.2018.00149

21. Aggarwal R, Farag S, Martin G, Ashrafian H, Darzi A. Patient perceptions on data sharing and applying artificial intelligence to health care data: Cross-sectional Survey. *Journal Medical Internet Research*. Aug 26 2021;23(8):e26162. doi:10.2196/26162
