# Supplementary material for: Community views on the secondary use of general practice data: Findings from a mixed‐methods study
Source: Health Expect. 2024 Feb 15;27(1):e13984. doi: 10.1111/hex.13984 (PMC10869884; doi:10.1111/hex.13984)
Supplement: Supplementary file 5 — Supporting information. [file HEX-27-e13984-s004.docx]

**Appendix 5 Focus Group Participant Demographics**

| Characteristic | N |
| --- | --- |
| Gender | |
| Female | 11 |
| Male | 11 |
| Geographical area | |
| NSW | 7 |
| VIC | 7 |
| QLD | 4 |
| SA | 1 |
| WA | 3 |
| Age (years) | |
| <29 | 10 |
| 30-54 | 5 |
| 65+ | 7 |
| Highest level of education | |
| Secondary school | 7 |
| TAFE college | 6 |
| University degree | 7 |
| University post-graduate | 2 |
| Employment | |
| Working part time | 4 |
| Working full time | 11 |
| Retired | 3 |
| Unemployed - looking for work | 2 |
| Student | 2 |
