# Supplementary material for: Community views on the secondary use of general practice data: Findings from a mixed‐methods study
Source: Health Expect. 2024 Feb 15;27(1):e13984. doi: 10.1111/hex.13984 (PMC10869884; doi:10.1111/hex.13984)
Supplement: Supplementary file 6 — Supporting information. [file HEX-27-e13984-s005.docx]

**Appendix 6: Online Survey Participant Demographics**

| Characteristic | N (%) |
| --- | --- |
| Gender | |
| Male | 1,270 (48.8%) |
| Female | 1,322 (50.8%) |
| Non-binary | 2 (0.1%) |
| I prefer to describe* | 1 (0.0%) |
| I prefer not to say | 9 (0.3%) |
| Age | |
| Gen Z/iGen/Centennials (1996-2010) | 325 (12%) |
| Gen Y/Millennials (1977-2010) | 962 (37%) |
| Gen X (1965-1976) | 413 (18%) |
| Baby Boomers (1946-1964) | 573 (22%) |
| Traditionalists/Silent Gen (1928-1945) | 319 (12%) |
| Residential location | |
| Sydney | 536 (20.6%) |
| NSW other than Sydney | 290 (11.1%) |
| Melbourne | 508 (19.5%) |
| VIC other than Melbourne | 155 (6.0%) |
| Brisbane | 251 (9.6%) |
| QLD other than Brisbane | 262 (10.1%) |
| Perth | 218 (8.4%) |
| WA other than Perth | 60 (2.3%) |
| Adelaide | 147 (5.6%) |
| SA other than Adelaide | 43 (1.7%) |
| NT | 27 (1.0%) |
| TAS | 56 (2.2%) |
| ACT | 51 (2.0%) |
| Employment | |
| Full-time employed | 1,115 (42.8%) |
| Part-time employed | 546 (21.0%) |
| Unemployed | 104 (4.0%) |
| Home duties | 162 (6.2%) |
| Student/Training | 96 (3.7% |
| Retired | 474 (18.2%) |
| Unable to work (e.g., disability/Work Cover) | 87 (3.3%) |
| Highest level of education | |
| No formal qualifications | 50 (1.9%) |
| Year 10 or school certificate | 233 (8.9%) |
| Year 12 or leaving certificate | 409 (15.7%) |
| Trade/Apprentice | 160 (6.1%) |
| Other TAFE/Certificate | 633 (24.3%) |
| University degree/higher degree | 1,103 (42.2%) |
| I prefer not to answer/I am not sure | 16 (0.6%) |
| Combined household income | |
| $1 - $19,999 per year ($1 - $379 per week) | 101 (3.9%) |
| $20,000 - $39,999 per year ($380 - $769 per week) | 375 (14.4%) |
| $40,000 - $59,999 per year ($770 - $1149 per week) | 334 (12.8%) |
| $60,000 - $99,999 per year ($1150 - $1919 per week) | 601 (23.1%) |
| $100,000 - 124,999per year ($1920 - $2399 per week) | 320 (12.3%) |
| $125,000 - $149,999 per year ($2,400 - $2879 per week) | 238 (9.1%) |
| $150,000 - $199,999 per year ($2880 - $3839 per week) | 231 (8.9%) |
| $200,000 or more per year ($3840 or more per week) | 168 (6.5%) |
| I prefer not to answer/I am not sure | 236 (9.1%) |
