# Supplementary material for: Community views on the secondary use of general practice data: Findings from a mixed‐methods study
Source: Health Expect. 2024 Feb 15;27(1):e13984. doi: 10.1111/hex.13984 (PMC10869884; doi:10.1111/hex.13984)
Supplement: Supplementary file 7 — Supporting information. [file HEX-27-e13984-s007.docx]

**Appendix 7: Participant responses to Conditions of Sharing (Trust, Transparency, Anonymity)**
